# Supplementary material for: Are carbon emissions trading and green financial instruments synergistic? -Comprehensive quantitative research based on content analysis
Source: PLoS One. 2024 Mar 7;19(3):e0298601. doi: 10.1371/journal.pone.0298601 (PMC10919601; doi:10.1371/journal.pone.0298601)
Supplement: S1 Data — (ZIP) [file pone.0298601.s001.zip › Data/地级市Z_F工作报告.zip!/░▓╗╒/╗┤─╧╩╨/└┤╫╘╣1⁄2╓┌║┼í╛┬φ┐╦╩2╛▌═°í┐_2017.docx]

各位代表：

现在，我代表市人民政府，向大会报告工作，请予审议，并请市政协委员和其他列席人员提出意见。

一、奋发有为，实现“十三五”良好开局

刚刚过去的2016年，是淮南决战决胜全面小康、奋力在资源型城市转型发展中闯出新路的重要一年。全市人民在省委省政府和市委的坚强领导下，全面贯彻党的十八大和十八届三中、四中、五中、六中全会精神，深入贯彻习近平总书记系列重要讲话特别是视察安徽重要讲话精神，认真落实省市第十次党代会要求，启动五大发展行动计划，千方百计稳增长、促改革、调结构、惠民生、防风险，经济社会保持平稳健康较快发展。

——经济运行稳中有进。初步核算，全市地区生产总值945亿元，增长6.5%;规上工业增加值343亿元，增长7%;财政收入150.9亿元，增长6.5%;社会消费品零售总额505亿元，增长10.5%；固定资产投资960亿元，增长5%，发展质量和效益稳步提高。

——经济结构更加优化。预计，三次产业结构为12.3：46.7：41，服务业占比提高1.5个百分点。非煤工业增加值占规上工业增加值的比重提高2.5个百分点，民营经济增加值占GDP比重提高0.5个百分点，万元GDP能耗下降3.5%，服务业投资占比提高8.7个百分点，产业向中高端不断迈进。

——项目建设卓有成效。全市瞩目的安理大新校区、职教园区一期相继建成，凤台淮河二桥、孔李淮河大桥建成通车，城市水系综合治理、采煤沉陷区综合治理项目取得积极进展，获批省级大数据产业集聚发展基地，智慧谷、新能源研究中心等重大创新平台投入使用，经济内生动力加速集聚。

——发展活力持续释放。新增各类市场主体23914家，增长29.7%，新增“四上”企业151家。新增农民专业合作社174个、家庭农场57个、土地流转5.5万亩。授权发明专利460件，万人有效专利拥有量3.92件，国家新型智慧城市和省级创新型城市创建步伐加快。

——民生福祉持续增进。13大类民生支出180.8亿元，占全市财政支出的86.1%。民生工程建设获得全省绩效奖补。实现34160人脱贫、48个贫困村出列。全市城镇新增就业5.4万人，农村转移劳动力3.74万人。城镇、农村常住居民人均可支配收入分别达27900元和10848元，增长6%、7%，城乡居民幸福感不断提升。

一年来，主要做了以下七个方面工作。

（一）聚焦综合施策，经济发展取得新成效。以推进供给侧结构性改革为主线，积极适应新常态，着力稳增长、打基础、增后劲。加快推进供给侧结构性改革。制定“1+6”实施方案。关闭退出3对煤矿、495万吨产能，妥善安置职工2.9万人。设立40亿元土地储备基金，统筹推进关闭矿井土地收储和人员安置。强化房地产去库存,全市商品房待售面积同比减少94.4万平方米。制定减轻企业负担26项措施，直接为企业减负19亿元，企业主营业务成本占比下降8.5个百分点。全年新增贷款85亿元，直接融资208.5亿元。新增“新三板”挂牌企业1家，38户企业在省股权交易中心成功挂牌。强化实体经济帮扶。出台促进经济持续健康较快发展实施意见等系列政策。产业发展基金投入实体经济10.4亿元。完善精准帮扶重点工业企业制度，研究解决180家企业涉及的土地、规划等589个问题，兑现财政支持工业经济发展奖补资金4693万元，全市规上工业企业利润总额增长110%，产成品库存下降8%。着力扩大有效投资。深入实施项目建设攻坚年活动，建立健全项目谋划推进机制，不断提高项目开工率、竣工率、达产率和转化率。全年亿元以上省重点项目新开工106个、续建131个、竣工61个，累计完成投资450亿元，完成年度计划的122.9%。工业、服务业亿元以上项目分别完成投资140.5亿元、254.6亿元。民间投资增长5.1%。争取中央预算内和省级补助资金2.45亿元。设立政府和社会资本合作（PPP）模式前期服务引导基金。积极推进开放合作。合淮同城化迈出坚实步伐，加快推进合淮工业走廊规划修编，与合肥市政府达成新桥国际产业园托管意向，寿蜀现代产业园落地项目20余个，济祁高速公路、206国道淮南段改造等合淮重要通道全面完工。招商引资取得新成效，新签约10亿元以上项目25个、总投资508.9亿元。实现进出口总额3亿美元，引进内资720亿元，实际到位外商直接投资2.6亿美元。不断夯实园区平台。省级以上园区累计完成固定资产投资236亿元。深入推进市经开区全员聘任聘用制改革，编制完成现代煤化工产业园总体规划，市高新区争创国家级高新区验收完毕，其他园区基础设施更加完善。

（二）聚焦结构调整，产业升级迈出新步伐。围绕构建创新型产业体系，累计下达产业发展基金扶持计划23.5亿元。支柱产业优化提升。坚持煤电一体、清洁发展，煤炭、电力行业规上工业增加值分别增长9.5%、5%。帮助煤炭企业减负脱困，启动“三供一业”分离移交工作，拨付失业保险调剂金1000万元，缓缴社会保险费9.7亿元，分别下调养老保险、失业保险企业缴纳比例1个、0.5个百分点。支持煤炭企业加快转型，淮南矿业集团、中煤新集公司扭亏为盈。主导产业加快成长。一以贯之推动中安煤化一体化项目建设，完成项目全面复工各项准备工作。现代装备制造业升级提速，首批新能源智能旅游房车顺利下线。新一代信息技术产业迅速发展，大数据产业实现产值26亿元，116家大数据关联企业录入国家统计基本单位名录库。出台加快电子商务发展的意见，寿县荣获“国家电子商务进农村综合示范县”称号。高成长性产业加快培育。累计完成投资419.5亿元，增长50%。服务业增加值增长7%。文化产业增加值增长10.5%，旅游收入和旅游人次分别增长17%、15%。建成并网光伏、风力发电项目装机容量42万千瓦。现代医药产业增加值增长7%。全年粮食总产284.8万吨，实现农产品加工产值233亿元。

（三）聚焦改革创新，经济社会增添新活力。落实全面深化改革和创新发展要求，进一步破除影响发展的体制机制障碍。稳妥推进重点领域改革。全年实施改革任务159项。实施“放管服”改革，构建“五清单一办法”权力运行管理机制。深化行政体制改革，完成不动产统一登记改革，稳步推进事业单位分类改革。深化国资国企改革，组建市融资担保集团，健全融资平台法人治理结构。深化商事制度改革，率先实施“五证合一、一照一码”，推广“双随机、一公开”。深化财税体制改革，全面推开“营改增”试点，政府债务分类纳入预算管理，债务规模总体可控。抓好农村金融综合改革，稳步推进凤台农村承包土地经营权抵押贷款试点。提前完成农村土地确权登记颁证试点工作。积极实施创新驱动发展战略。设立1亿元天使投资基金，全社会研发经费投入14.5亿元。战略性新兴产业产值、高新技术产业增加值分别达49亿元、22亿元。制（修）订国家、行业、地方标准6项。获批院士工作站2家、省级博士后工作站1家，净增高新技术企业14家，新增科技企业孵化器7家、众创空间7家、创新团队10家，引进高层次人才212人。

（四）聚焦统筹协调，城乡建设得到新加强。遵循“一尊重、五统筹”理念，加快城乡一体化发展。“多规合一”试点全面推进。实施淮南“多规合一”规划方案，盘活建设用地66平方公里，解决了我市规划、土地、建设相互冲突问题。寿县“多规合一”信息平台上线运行，审批效率得到极大提升。区划调整效果显著。寿县划入拓展了淮南城市发展空间，优化了全市生产力布局，提高了城市综合承载能力。全市上下坚持对寿县高看一眼、厚爱三分，积极谋划推进综合交通、产业布局、园区共建和文旅整合，加快“南工北旅生态县”建设步伐，实现了寿县与全市的融合发展。凤台县与八公山区部分区域区划调整顺利完成，凤台跨河发展优势更加凸显。城乡建设扎实推进。山南新区建设迈出新步伐，全年完成投资100亿元，实验中学新校区、市级公租房等项目建成投用，管网、公交等配套服务体系进一步完善。老城提升稳步实施，维修改造老旧小区39个，启动城区84个菜市场改造升级，新改建公厕129座，整治小街小巷80条、住宅小区228个。保障性安居工程开工建设5092套，基本建成6508套。采煤沉陷区搬迁安置房开工建设4500套，搬迁入住4400户。实施朝阳西路等7条道路建设，完成10条主城区20米以下道路中修。新改建各类管网120公里。美丽乡村建设扎实推进，基本完成27个乡镇政府驻地建成区整治、25个省级中心村建设。获批全国重点镇4个、中国少数民族特色村寨1个。完成危房改造3800户，解决9.63万农村人口安全饮水问题。加快推进西淝河沿淮洼地治理、大型泵站更新改造等水利工程。实施10条国省干线公路改造，改造农村公路941公里。城市创建深入开展。合力推进“六城同创”。李元芳等3人当选中国好人。信用体系加快建设。完成中北巴士股权回购，新辟调整公交线路10条，更新公交车306辆。建成公共自行车系统项目。拆违征迁378.3万平方米。推进城区环境卫生综合整治，城市形象得到提升。

（五）聚焦绿色发展，生态环境实现新优化。深入贯彻绿色发展理念，严格环保执法，进一步改善环境质量。加强环境建设。节能减排实现年度目标。扎实推进大气污染治理，完成工业大气污染治理项目44个，黄标车淘汰、燃煤小锅炉、物料堆场等整治工作进展顺利。午秋两季秸秆禁烧实现卫星监测“零火点”。全年空气质量优良天数比率为79%。扎实推进全国水生态文明试点市创建，开展黑臭水体、入淮排污口整治，加快湖泊湿地生态保护，淮河干支流断面水质达标。加大非法采砂打击力度，淮河河道管理成效显著。实施节能与资源综合利用项目20个，开展省建筑垃圾处置及再生资源化利用试点。全面整改省环保督察检查反馈问题。加强采煤沉陷区综合治理。完成采煤沉陷区综合治理规划中期修订。建立沉陷区“一张图、一张表、一个数据库”动态管理机制。争取国家治理资金33.4亿元，实施一批沉陷区综合治理项目。深入推进关闭矿井生态修复、废弃煤矿矿山地质环境治理。加强生态屏障建设。扎实开展国家森林城市创建，成片造林4.2万亩。实施焦岗湖国家湿地公园环境治理项目，加强八公山、舜耕山、上窑山景区管理，着力打造城市“绿肺”。完成一批城市公园、街头游园、城市绿道建设，不断拓展群众休憩空间。创建省级生态乡镇3个、生态村6个，农村生活环境得到优化。

（六）聚焦社会民生，人民生活得到新改善。深入贯彻共享发展理念，不断强化民生兜底责任。脱贫攻坚全面推进。全年投入4.3亿元财政资金用于扶贫开发，顺利完成年度目标任务。实施“4+4”脱贫攻坚工程和扶贫行动计划，制定扶贫项目管理、脱贫攻坚监督检查等7个办法，建立“1+18”脱贫攻坚制度体系，扶贫保障措施更加完善。就业创业形势向好。城镇登记失业率4%。新增发放创业担保贷款3.45亿元，发放稳岗补贴1.2亿元。开展就业创业培训3.2万人。劳动合同签订率99.8%。社会保障体系更加完善。实现各类参保416万人次。企业退休人员养老金、失业保险、城镇居民基本医疗保险、城镇低保、农村低保、农村五保分散供养和社会散居孤儿保障标准分别提高8.2%、49.7%、5%、13.6％、32.5％、43%和16.6%，实现低保线和脱贫线“两线合一”。社会事业稳步发展。优先发展教育事业，扎实做好4个县区迎接国家义务教育发展基本均衡县区认定工作，全面完成第二期学前教育三年行动计划。持续深化综合医改，着力破除以药养医机制，智慧医疗入围国家财政部第三批PPP示范项目。有序实施全面二孩政策，人口自然增长率7.86‰。文艺创作精品不断涌现，一批项目入选省“四个一”项目、文化部重点文化产业项目库。成功举办淮南国际半程马拉松赛、凤台中国警营半程马拉松赛。援藏援疆、地震、气象、档案、民族宗教和地方志工作不断加强，妇女儿童、老龄、残疾人和红十字事业取得新进展。社会治理创新步伐加快。推进平安淮南建设，建立完善数字化立体化治安防控体系，完成社会管理服务信息化平台“一中心三系统”建设。积极开展信访领域专项整治，信访总量大幅下降。深入开展“铸安”行动，安全生产形势总体稳定。积极推广网上阳光村（居）务经验。全面开展食品药品规范管理示范单位创建。扎实推进国防教育、国防动员工作，军民融合发展取得新成效，荣获“全国人民防空先进城市”称号。

（七）聚焦作风建设，政府治理能力实现新提升。牢固树立“四个意识”，锲而不舍推进政府自身建设。不断加强作风建设。扎实开展“两学一做”学习教育、“讲看齐、见行动”学习讨论、优化发展环境年活动，深入开展小金库、招投标等专项整治。严格落实中央八项规定，持续深化作风建设。全市“三公”经费支出下降29.7%，公务用车制度改革节支2688万元。建立市县统一的公共资源交易平台。市长热线办理群众来电10万余件，回复网民留言2700余条，荣获人民网网民留言办理工作10周年贡献奖。扎实推进依法行政。依法执行人大决议决定，积极推进民主协商，办理人大代表议案建议281件、政协提案366件，提请审议地方性法规2件。制定政府规章2件。完成重大事项合法性审查185件，依法办理行政复议案件，积极应诉行政诉讼案件。实行政府系统新任职人员宪法宣誓制度。推进制度规范全覆盖，编制实施年度制度建设清单。全面贯彻落实中国共产党党组工作条例、党内政治生活准则、党内监督条例等党内法规，制定市政府党组工作规则。着力强化行政监督。推进审计监督全覆盖，建立审计问题“三单合一、整改销号”制度，全年审计（调查）单位934家，促进增收节支4.1亿元。强化行政监察，给予政纪处分291人，不断完善党风廉政和反腐败工作制度体系，进一步提升政府执行力和公信力。

各位代表！在错综复杂的宏观环境下，在不确定因素较多的经济背景下，这样的成绩来之不易。这是市委坚强领导、市人大依法监督、市政协民主监督的结果，也是全市广大干群坚定信心、同心协力、奋力拼搏的结果。在此，我代表市人民政府，向全市人民，向驻淮部队、武警官兵和政法干警，向各民主党派、各人民团体和社会各界人士，向所有关心支持淮南改革发展的海内外朋友们，表示衷心感谢和崇高敬意！

在肯定成绩的同时,我们也清醒地看到前进中的困难和挑战。一是资源型城市转型发展探索艰难、任重道远，接续替代产业发展不足，项目支撑作用较弱，民营经济、县域经济、园区经济发展不充分，经济企稳回升的基础需要夯实。二是民生保障能力和水平有待提高，城乡基础设施欠账较多，教育、医疗、社会保障等领域存在不少短板。三是发展环境仍需优化，生态环境建设任务繁重。四是政府治理体系和治理能力现代化水平亟待提升，少数干部缺乏担当、作风不实。对此，我们将采取更加有力的措施，切实加以解决，决不辜负全市人民的期望。

二、开拓创新，扎实做好2017年各项工作

今年是实施“十三五”规划的重要一年，是推进供给侧结构性改革的深化之年，做好今年工作意义重大。当前，淮南发展仍处于可以大有作为的重要战略机遇期，我们必须牢牢扭住发展第一要务，保持战略定力，抢抓发展机遇，坚定不移落实新发展理念，以优异成绩迎接党的十九大胜利召开。今年工作的总体要求是：全面贯彻党的十八大和十八届三中、四中、五中、六中全会精神，深入贯彻习近平总书记系列重要讲话特别是视察安徽重要讲话精神，统筹推进“五位一体”总体布局，协调推进“四个全面”战略布局，坚持稳中求进工作总基调，牢固树立和贯彻落实新发展理念，适应把握引领经济发展新常态，坚持以提高发展质量和效益为中心，贯彻落实“五大政策”，以推进供给侧结构性改革为主线，以增进人民福祉、促进人的全面发展为出发点和落脚点，坚持“1235”发展思路，实施五大发展行动计划，全面做好稳增长、促改革、调结构、惠民生、防风险各项工作，不断开创“五大发展”的美好淮南建设新局面。

经济社会发展的主要预期目标是：地区生产总值增长7%以上，财政收入增长6%以上，固定资产投资增长10%左右，社会消费品零售总额增长10%，城镇和农村常住居民人均可支配收入分别增长7%以上、8%以上，城镇登记失业率控制在4%以内，节能减排完成省下达的目标任务。

做好今年工作，要把握好以下四个原则。

一是坚持稳中求进工作总基调，切实保持全面建成小康社会战略定力。深刻理解、准确把握中央关于稳中求进工作总基调的重要论述，牢固树立问题导向和底线思维，坚定信心，保持定力，以进促稳，稳预期、稳政策、稳大局，在发展动力、发展布局、发展关系、发展保障等方面探索淮南路径、编制淮南方案，确保如期全面建成小康社会。

二是坚持以新发展理念为指引，适应把握引领经济发展新常态。坚定地用新发展理念统领发展全局，深化对发展规律的认识和把握，抓住发展战略机遇期，盯紧转型升级窗口期，瞄准产业提升拐点期，不断探索新方法、闯出新路子，在新常态的变革大潮中赢取未来。

三是坚持以推进供给侧结构性改革为主线，加快培育经济增长新动能。紧紧围绕供给侧结构性改革的最终目的、主攻方向和根本途径，结合淮南实际和需要，做好供给“加减乘除法”，提升供给质量。正确处理政府和市场关系，通过改革优化要素市场，释放各类主体活力。强化创新驱动作用，推动“双创”向纵深发展，促进新动能发展壮大、传统动能焕发生机。

四是坚持全面实施五大发展行动计划，奋力在资源型城市转型发展中闯出新路。坚决落实省委市委决策部署，逐项对标、分类攻坚、精准施策，把行动硬化到具体项目上、体现在发展支撑上。坚持转型发展不动摇、一张好的蓝图干到底，围绕打好“四张资源牌”，加快产业转型、生态转优、机制转轨，拓展发展路径，优化发展结构，厚植发展优势，乘势而上闯出转型发展新路。

今年重点抓好以下八个方面工作。

（一）更加注重供给侧结构性改革，加快新旧动能转换

落实“三去一降一补”任务，推进农业供给侧结构性改革，着力促进实体经济稳定健康发展。

积极化解过剩产能。坚定不移完成煤炭行业去产能年度任务，推进关闭矿井土地收储，稳妥做好职工分流安置工作。加快国有企业职工家属区“三供一业”分离移交，帮助企业轻装上阵、加快发展。抢抓去产能机遇，运用法治化、市场化手段，做好融资协调工作，多渠道化解企业债务。支持淮南矿业集团、中煤新集公司多元发展，帮助企业实现近期解困、远期转型。支持宏泰钢铁重组升级、技术改造、做大做强。

促进房地产市场平稳健康发展。以非住宅商品房去库存为重点，统筹商品住房去库存，鼓励开发企业将库存的商业地产改造为科技企业孵化器和众创空间。加强市场监管，引导住房消费，强化房地产用地供应管理，逐步实现供求基本平衡。把去库存和促进人口城镇化结合起来，深化户籍制度改革，鼓励农民进城购房，加快农业转移人口市民化。继续实施促进房地产市场健康发展政策，扩大棚改货币化安置比例，加快公租房分配，培育住房租赁市场。

着力振兴实体经济。围绕防风险推进去杠杆，优化融资结构，推进企业上市融资，扩大“4321”新型政银担合作试点和“税融通”贷款业务量，降低企业杠杆率。落实降成本减轻实体经济企业负担实施意见，在减税、降费上加大工作力度，打好降低制度性交易成本、用能用地成本、物流成本等组合拳，多渠道减轻企业负担，助推企业增效和实体经济发展。大力弘扬企业家精神和工匠精神，加强品牌建设，强化质量管理，引导企业形成独有的比较优势。

大力推进补短板。加快补齐县域和民营经济发展短板。实施县域经济提升行动计划，完善分类考核办法，培育省级产业集群专业镇，打造各具特色的经济板块。实施民营经济提升工程，支持本土民营企业发展，新增民营企业4500家、个体工商户10000家，培育省级“专精特新”中小企业12家。民营经济增加值占GDP比重提高1个百分点以上。推动制度性和体制性改革，加快补齐制度短板。在创新能力、产业发展、基础设施、生态文明建设等方面实施一批补短板重点项目。

加快推进农业供给侧结构性改革。把增加绿色优质农产品供给放在突出位置，实施生态农业产业化示范创建工程。加快构建现代农业生产体系，推进高标准基本农田建设和永久性基本农田划定，加快水稻、水产、水利融合发展，建设粮食绿色增产模式攻关示范区，多措并举稳定粮食产量。加快构建现代农业产业体系，壮大发展优势特色产业，加强休闲农业与乡村旅游示范点建设，着力打造粮食深加工、畜禽水产养殖业加工、地方名优农产品加工三大产业链。加快构建现代农业经营体系，发展多种形式适度规模经营，创建市级示范农民专业合作社30家、家庭农场50家。实施绿色兴农重大工程，强化农产品质量安全监管，认证“三品一标”20个。推进“智慧粮食”仓储信息化，加快中储粮淮南直属库、农业科技园、农产品加工园和物流园建设。

（二）更加注重产业升级，加快提升经济发展质量效益

坚持工业强市不动摇，用好产业发展基金，全面打好“四张资源牌”，着力构建创新型现代产业体系。全年净增规上工业企业、限上商贸企业各60家，规上工业增加值、服务业增加值分别增长7%、10%。

打优煤电资源牌。大力发展煤电联产、煤化联产、煤气联产，鼓励煤炭企业和发电企业通过控股或参股等形式一体化发展，推动煤炭产业向清洁高效安全利用转变、向价值链高端延伸，不断提升煤电产业的整体实力和综合竞争力。加快中安煤化一体化项目建设，促进淮化、德邦等传统化工企业技术改造，推动新型煤化工产业稳定增长。

打强数据资源牌。加快完善大数据存储体系、交易体系和应用服务体系，促进以大数据为代表的新一代信息技术产业加速成长。加快与中国科学院大气物理研究所、中国航天系统科学与工程研究院等战略合作，推动数据中心、行业应用超算中心、大数据应用平台、大数据加工场、钱学森智库淮南中心等重点工程建设。充分发挥华东区域最大的数据存储中心、高带宽的信息高速出口、卫星数据等资源优势，结合淮南各类数据应用平台，推动省级大数据产业集聚发展基地建设，积极争创国家大数据中心的区域中心。

打好科教资源牌。依托国家级科研院所、研发平台，加快科研成果就地转化，着力打造一批高端产业。实施创新人才和企业家培育集聚工程，培养引进200名高端人才、10家创新团队，新增专业技术人才8000名。充分发挥在淮高校、科研院所优势，深化产学研用合作，支持组建产业技术创新联盟，推进优质科教资源与主导产业、高成长性产业有效嫁接、加速融合。发挥职教集团协调创新作用，加强职业教育和技能培训，培养高技能人才5000名，积极参与技工大省建设。实施《中国制造2025安徽篇》淮南推进工程，大力扶持龙头企业发展，加快建设现代装备制造、现代医药市级战新基地。

打响文化资源牌。实施文化旅游强市建设工程，挖掘整合地域特色文化，打造文化产业集群。推动文旅融合发展，高标准编制旅游总体规划，规划建设文化旅游生态长廊，重点打造“一山（八公山）一湖（焦岗湖）一古城（寿州古城）”淮南旅游品牌。推进八公山景区整体建设，支持寿州古城·八公山、焦岗湖创建国家5A级景区。促进文化旅游业与其他业态深度融合，打造旅游精品线路。加强旅游市场营销、旅游行业规范。探索实施乡村旅游后备箱行动、旅游服务标准化工程。文化产业增加值增长10%以上，旅游收入、旅游人次分别增长16%、15%。

（三）更加注重重大项目建设，加快夯实经济社会发展基础

落实“四督四保”要求，围绕全局性、基础性、战略性重大工程项目建设，加快培育新的经济增长点。

继续开展项目建设攻坚年活动。全年完成固定资产投资1000亿元以上，其中工业投资320亿元。计划开工亿元以上省重点项目110个、续建144个、竣工30个、储备160个。新开工光电产业园、贵州益佰医药产业及孵化基地、航天体验园等一批产业类项目，引江济淮淮南段、正峡段行洪区调整改造、瓦埠湖大桥、玉兰大道等一批基础设施类项目，淮西湖生态修复、技工学校新校区、舜新家苑康养中心等一批社会事业类项目，谋划建设城市轻轨、航天卫星综合应用产业园、新桥机场快速通道等一批重大牵动性项目。

强化项目建设保障。加强制度保障，完善市级领导联系重大项目、项目谋划推进等机制，严格督查通报和考核奖惩，全方位推动项目建设提速。加强用地保障，强化土地供应计划管理，积极盘活闲置土地。加强资金保障，积极对接国家专项建设基金，运用好政府和社会资本合作（PPP）模式，鼓励金融机构扩大项目贷款比重、信贷支持重点向工业项目倾斜。市建发集团、产发集团分别计划融资175亿元、30亿元。

（四）更加注重创新发展，加快激发经济发展活力动力

实施创新体系建设一号工程，系统推进“全创改”、“三重一创”建设，不断释放改革创新红利。

提升创新能力。加强新型智慧城市顶层设计，加快构建技术和产业、金融和资本、平台和企业、制度和政策四大创新体系。用好各类天使基金、创业投资基金和股权投资基金，深入推进创新创业。新增高新技术企业10家，培育企业技术中心10家、“科技小巨人”企业6家。大力推进“两化融合”，建设“互联网+智慧企业”100家。实施质量品牌升级工程，培育中国驰名商标2件。加强知识产权保护和运用，申请专利2000件。

建设创新平台。发挥好江淮云、智慧谷、市经开区信息产业园、寿县“互联网+”产业园等平台作用，加快推进“1+3+8”科技企业孵化器体系建设，建成国家煤化工产品质检中心二期，组建标准化研究院。积极培育省级以上重点实验室和省级工程技术研究中心，新增院士工作站1家，建设“双创”示范基地2个。推进“宽带淮南”建设，不断提高固定宽带用户普及率、移动宽带用户普及率。

全面深化改革。深化“放管服”改革，推动行政审批制度改革，探索行政许可权相对集中改革，加快行政审批标准化建设，动态优化“五个清单”，强化权力运行事中事后监管。深化行政体制改革，推进环保、审计等体制改革。大力推进国资国企改革，支持淮南矿业集团整体上市。持续推进新型城镇化试点、投融资体制、财政金融体制、国税地税征管体制改革。深化商事制度改革，扩大“证照合一”覆盖面，建立严重违法失信企业名单管理和联合惩戒机制。积极推进农村综合改革，实施农村土地所有权、承包权、经营权分置改革，开展农村集体资产股份合作制、土地经营权担保试点。推进供销社综合改革。深化综合医改，加快完善分级诊疗制度。

（五）更加注重协调发展，加快推进城乡一体化建设

牢固树立城乡协调发展理念，强化城市规划、建设和管理，推进“四创一建”，加快“六城同创”，努力构建城乡区域协调发展新格局。

坚持规划先行。推进“多规合一”平台建设。完成城市总体规划、土地利用总体规划修编，启动淮南市控规单元规划、重要地段城市设计编制。完成城市综合服务设施规划、八公山风景区总体规划、县域乡村建设规划和美丽乡村规划编制。

推动区域协调。统筹新区老区建设，实施现代综合通道建设工程，优化区域空间布局和规模结构，全面提升城市能级。着力完善山南新区综合配套，加快道路、医院、学校、酒店、商场等建设。着力提升老区形象，扎实开展“两治三改”三年专项行动，推进谢家集、八公山老工业区整体搬迁改造。开工保障性安居工程2660套、基本建成1873套，新开工沉陷区安置房3800套、搬迁入住4600户。完成28个老旧小区改造。着力完善基础设施，规划建设广场、步行道等公共活动空间。实施城市排水防涝三年行动计划，推进“海绵城市”建设。着力畅通城市内循环，加快跨区域快速通道和城市主次干道建设，加密城市路网。

注重城乡协调。持续扩大城乡消费，加快电商淮南建设，推进省级服务业集聚区建设，建成一批专业市场。建立健全城乡统筹机制，积极推进城乡教育、医疗、卫生等基本公共服务一体化。健全美丽乡村建设长效机制，启动31个乡镇政府驻地建成区整治、55个省级中心村建设，完成危房改造年度任务，扎实开展农村环境综合整治。深入实施农村道路畅通工程，改造县乡道路155.5公里、村级道路313.4公里。加强水利“一规四补”建设。实施农村饮水安全巩固提升、小型水利改造提升工程。推进农村电网改造升级。加快建设公交、客货运站场，投放新能源公交车200辆，推进城乡公交一体化。

加强城市管理。实施文明淮南建设工程，深化拓展群众性精神文明创建活动。探索建立长效机制，巩固文明城市创建成果。推进城市管理执法体制改革试点，实行“建管分离”。全面启动客运站点周边、公路铁路河流沿线、城市出入口等重点区域环境综合整治。开展车辆乱停乱放、户外广告等专项整治，加强城市停车场建设，完成数字城管升级改造。创新出租车经营服务管理。强化环卫作业市场化运营，实施一批生活垃圾处理项目。加强征迁拆违，落实征迁资金和安置房源，征迁拆违500万平方米以上。

（六）更加注重绿色发展，加快推进生态文明建设

坚持生态优先、绿色发展，努力在更高层次上实现绿水青山与金山银山的统一，打造生态文明建设的淮南样板。

筑牢生态安全屏障。全面落实主体功能区规划，划定并严守生态保护红线。推进国家森林城市创建，完成成片造林7000亩以上，加强淠河生态经济带建设，保护好“三山”、“三水”等重要生态屏障。加强港口岸线使用管理、湿地管理和综合开发利用，推进关闭矿区、非煤矿山生态修复。争取国家重点采煤沉陷区综合治理试点区政策，加快重点地区生态环境治理。实施园林绿化精品示范工程，推进城市公园、绿道等建设，积极谋划煤矿遗址公园。

加强环境综合治理。深入实施大气、水、土壤污染防治行动计划。开工建设大气环境监测与综合治理系统项目。扎实开展建筑扬尘、非法堆场、农村燃煤小锅炉、黑加油站等专项整治，做好秸秆禁烧和综合利用工作。实施工业污染源全面达标排放计划，完成燃煤发电机组超低排放改造和黄标车淘汰任务。继续提升空气质量优良天数比率。全面推行河长制，加强水功能区管理和饮用水水源地达标建设，开展盗采地下水专项整治，加强城市黑臭水体、龙王撇洪沟等5条水系治理。建立雨污分流城镇污水系统，建成首创第一污水处理厂二期，所有工业园区建成污水集中处理设施。抓好土壤和农业面源污染防治。

构建绿色发展模式。争创国家生态文明建设示范区、国家资源型城市可持续发展示范区。大力发展节能环保产业，支持传统产业清洁化生产，加强煤矸石、粉煤灰、矿井水等综合利用，开发利用生物质能，推动绿色建筑发展。全面普及生态文明教育，倡导文明、节约、绿色的消费方式和生活习惯。

完善生态文明制度。建立健全自然资源资产产权制度、国土空间用途管制制度、资源有偿使用和生态保护补偿机制，推行合同能源管理和环境污染第三方治理。坚持源头严控、过程严管、后果严惩，强化生态环境监测预警，健全企业环境信用记录和违法排污黑名单制度，严格落实建设项目“三同时”制度。探索实行领导干部自然资源资产管理和环境保护责任离任审计。

（七）更加注重开放发展，加快培育经济发展新优势

持续扩大对外开放，不断释放发展新潜能，培育发展新优势，着力打造内陆开放新高地。

积极推进合淮同城化。办好合肥都市圈城市党政领导第八次会商会议，加速与合肥全方位等高对接。坚持规划协同、交通协同、产业协同、社会事业协同，积极参与合肥都市圈“十三五”总体规划和专项规划编制，争取合淮工业走廊规划获批。深化与合肥合作共建寿蜀现代产业园、新桥国际产业园，谋划建设新桥空港物流园，推进科教、医疗、社保等资源合作共享。

实施园区转型升级工程。壮大国家级经开区，加快产城一体建设，当好全市转型发展的排头兵。紧抓市高新区创建国家级高新区机遇，以创促建，建设“四中心一基地”，促进高新技术产业集聚发展。进一步加快现代煤化工产业园基础设施建设，积极做好防护距离范围内居民搬迁安置工作，为项目落地创造条件。完善园区转型发展支持政策，推进各类产业园竞相发展。

努力扩大招商引资。注重精准招商、产业链招商、以商招商、小分队招商，强化工业、制造业招商。推行招商引资项目责任制，着力提高签约项目的落地率，确保引进内资、引进省外亿元以上项目资金额和外商直接投资平稳较快增长。进一步优化投资环境，完善招商引资政策，落实招商引资项目审批服务“交钥匙”工程，积极构建“亲”“清”新型政商关系。

不断提升对外开放水平。积极融入国家“三大战略”，推进开放大平台、大通关建设，构建开放型经济新体制。参与淮河生态经济带、中原经济区建设，加快建设淮南港，谋划打造区域性综合物流集散中心。实施外贸主体培育壮大工程，推进六大出口基地、外贸综合服务平台建设，加快海关特殊监管区域、跨境电商综合试验区申报。建立口岸工作联席会议制度，推动设立出入境检验检疫正式机构。支持企业“走出去”，加强境外投资、工程承包、劳务技术合作。密切友城交流，助推对外开放。

（八）更加注重共享发展，加快筑牢民生保障安全网

统筹推进各项社会事业发展，高标准实施好省级民生工程，顺应人民群众对美好生活的向往。

扎实推进脱贫攻坚。实施四大脱贫攻坚工程、四大扶贫行动计划，重点推进产业扶贫、就业扶贫、健康扶贫、教育扶贫、金融扶贫，增强贫困地区自我发展能力。坚持领导干部深入贫困村、贫困户常态化机制，广泛凝聚社会力量，全年完成3万人脱贫、50个贫困村出列任务。实施“优先发展”提升行动，加快少数民族和民族聚居地区经济社会发展。

扎实推进就业创业。以创业带动就业，继续实施“创业淮南”行动计划、高校毕业生就业创业促进计划，加快推进创业服务云平台建设，新增发放创业担保贷款2亿元，组织创业培训3000人。全年城镇新增就业4.2万人，农村转移劳动力3.7万人，确保零就业家庭动态“清零”。

扎实推进教育提质。实施现代教育体系建设工程。大力发展学前教育，实施第三期学前教育三年行动计划。促进义务教育均衡发展，确保寿县通过国家义务教育发展基本均衡县认定。推进普通高中多样化发展。引导支持社会力量兴办职业教育。迁址新建特殊教育学校。推进智慧教育，加快教育信息化建设。

扎实推进文化服务供给。加快健全公共文化服务体系，不断完善以县区“两馆”、乡镇综合文化服务中心、农民文化乐园等为重点的公共文化设施网络。加强文物、非物质文化遗产保护，推进文化精品创作、文化惠民工程。繁荣发展哲学社会科学、文学艺术、新闻出版和广播影视事业。

扎实推进健康淮南建设。开展省级卫生城市创建。建成市妇幼保健院新院、全科医生临床培养基地，开工建设淮南市中医院新院。完善计划生育服务管理。推动全民健身和全民健康深度融合，促进公共体育设施向社会免费开放，举办市第八届运动会，每月举行一次群众性体育活动。完善食品药品安全治理体系，创建省级食品药品安全示范城市。

扎实推进社会保障体系建设。落实全民参保计划，做好社会保险登记工作。持续推进机关事业单位养老保险制度改革，加快整合城乡居民基本医疗保险制度。完善社会救助体系，推进养老服务业综合改革，努力打造“医养护康”四位一体养老模式。

扎实推进社会治理创新。深化社会稳定风险评估工作，推进市县乡村四级综治中心建设。完善社会治理体制机制，依法防范和严厉打击各类违法犯活动。深入开展安全生产“铸安”行动，加强综合防灾减灾救灾能力和应急体系建设。完成第十届村民委员会换届选举。支持工会、共青团、妇联等人民团体更好发挥桥梁纽带作用，做好外事侨务、对台、妇女儿童、全民科学素质、宗教和机关事务管理工作，加快人民防空、地震、气象、档案、残疾人和地方志等各项事业发展。推动军民深度融合发展，加强国防动员、兵役征集、国防教育和双拥工作，争创全省双拥模范城，不断巩固军政军民军地团结良好局面。

三、不忘初心，持续推进政府自身建设

毫不动摇坚持党的领导、加强政府系统党的建设，坚定不移“讲看齐、见行动”，加快政府治理体系和治理能力现代化，全面提高政府依法施政能力。

坚定政治定力，做到忠诚看齐。牢固树立“四个意识”特别是核心意识、看齐意识。坚定自觉讲看齐，向党中央看齐，向党的理论和路线方针政策看齐，向党中央决策部署看齐，确保中央、省市委决策部署落地生根。坚决维护习近平总书记党的领导核心地位，坚决服从大局，做到党中央提倡的坚决响应、党中央决定的坚决执行、党中央禁止的坚决不做。坚定理想信念，强化理论武装，增强“四个自信”，用理想之光照亮奋进之路，用信仰之力开创美好未来。

树立法治思维，推进依法行政。带头尊法学法守法用法，严守法律底线，自觉做到法定职责必须为、法无授权不可为。全面开展“七五”普法。坚持依法决策，健全重大决策程序，建立决策终身责任追究制度和责任倒查机制。积极推动重点领域立法，实行重大行政执法决定法制审核制度，推进行政执法全过程记录试点。自觉接受党内监督、人大法律监督、政协民主监督、社会舆论监督，认真做好人大代表议案建议和政协提案办理工作。积极推进民主协商，广泛听取各民主党派、工商联、无党派人士意见。全面推进政务公开，让权力在阳光下运行。

弘扬担当精神，坚持为民勤政。大力倡导敢于负责、勇于创新的担当精神，健全正向激励机制，探索容错纠错机制，鼓励和保护广大干部干事创业的积极性，努力用干部的辛苦指数换取人民群众的幸福指数。进一步健全为企业、群众和基层服务制度，加快“12345”政务服务直通车升级，推动“互联网+政务服务”改革，努力打造“7×24小时”“不打烊”的政务服务。健全督查问责机制，坚决整肃庸政懒政怠政行为。

坚守清廉本色，强化从严治政。时刻绷紧政治纪律和政治规矩这根弦，严格执行党内法规，加强和规范党内政治生活。认真落实全面从严治党主体责任，严格履行“一岗双责”，坚决实行“一案双查”，严厉惩治侵害群众利益的不正之风和腐败行为。严格遵守中央八项规定和省市相关规定，严控“三公”经费支出。强化行政监察和审计监督，着力打造为民务实清廉政府。

各位代表！新的一年充满新的希望，新的征程承载新的梦想。让我们更加紧密地团结在以习近平同志为核心的党中央周围，在中共淮南市委的坚强领导下，凝心聚力，开拓进取，奋力在资源型城市转型发展中闯出新路，为如期全面建成小康社会、加快建设“五大发展”的美好淮南而努力奋斗!
